# A cross sectional study to investigate internal hernia post left-sided colectomy preserving superior rectal artery

**DOI:** 10.1016/j.amsu.2019.10.026

**Published:** 2019-11-04

**Authors:** Tetsuro Taira, Koji Murono, Hiroaki Nozawa, Daisuke Hojo, Kazushige Kawai, Keisuke Hata, Toshiaki Tanaka, Soichiro Ishihara

**Affiliations:** Department of Surgical Oncology, The University of Tokyo, 7-3-1 Hongo, Bunkyo-ku, Tokyo, 113-8655, Japan

**Keywords:** Internal hernia, Stoma, Laparoscopic surgery, Left colectomy, Superior rectal artery, IMA, Inferior mesenteric artery, SMA, Superior mesenteric artery, SRA, Superior rectal artery

## Abstract

**Background:**

and Purpose: Intestinal obstruction caused by an internal hernia projecting through a mesenteric defect is a rare sequela of laparoscopic colectomy, as surgeons usually leave such defects open. In this study, we investigated cases of internal hernia after laparoscopic left-sided colectomy.

**Methods:**

Data of 308 patients who underwent laparoscopic left hemicolectomy or sigmoidectomy at our institute between 2013 and 2018 were retrospectively reviewed. Patient characteristics and surgical variables were analyzed. The distance between the superior rectal artery (SRA) and abdominal aorta at the level of aortic bifurcation was measured using postoperative computed tomography in patients who underwent SRA-preserving colectomy.

**Results:**

In all, 3 patients (0.97%), all of whom had undergone colostomy without anastomosis and with SRA preservation, developed internal hernia passing between the SRA and the aorta. The distance between the SRA and abdominal aorta in patients who underwent ostomy was significantly more than that in patients who underwent non-ostomy (10.6 mm vs. 4.7 mm, respectively, p < 0.001).

**Conclusions:**

SRA preservation and stoma construction are potential risk factors for internal hernia after laparoscopic left-sided colectomy. Lifting of the SRA due to stoma construction possibly enlarges the space between the SRA and aorta. When colostomy is created, it is important to evaluate the space behind the SRA.

## Highlights

• Internal hernia projecting through a mesenteric defect is a rare sequela of laparoscopic colectomy, as surgeons usually leave such defects open.
• However, leaving a residual defect may increase the incidence of internal hernia.
• In this study, we assessed the cases of internal hernia after laparoscopic colectomy.

## 1 Introduction

Several studies have documented better short-term outcomes of laparoscopic colectomy as compared to conventional open colectomy [[1], [2], [3], [4], [5]]. The advantages of laparoscopic colectomy include decreased pain, improved cosmesis, and shortened hospital stay [2,5]. Several reports have shown that laparoscopic colectomy is associated with a lower incidence of small bowel obstruction than open colectomy. The reported rates of postoperative small bowel obstruction after laparoscopic colectomy and open colectomy are 2.0%–7.8% and 3.0%–18.3%, respectively [1,[5], [6], [7]].

Postoperative intestinal obstruction is mainly caused by adhesions of the small intestine, but may rarely be caused by an internal hernia projecting through a post-colectomy mesenteric defect [8]. Unlike in open surgery, laparoscopic closure of a mesenteric defect is inherently challenging and inadvertent injury to the marginal vessels may compromise blood supply to the anastomosis [9,10]. Moreover, incomplete closure of the mesenteric defect may leave a narrow residual defect, which may actually increase the risk of internal hernia [11]. For these reasons, many surgeons leave the defect open during laparoscopic surgery [12]. However, according to Masubuchi et al. leaving a residual defect may increase the incidence of internal hernia [11]. In a recent meta-analysis, the incidence of internal hernia after laparoscopic colectomy was 0.65%; 64.3% cases of internal hernia occurred after left-sided resection [8].

Most of the internal hernia after laparoscopic colectomy was caused by the mesenteric defect, but Ichimura et al. reported a case of internal hernia through the mesenteric opening rimmed with the mesocolon and preserved superior rectal artery (SRA) after laparoscopic left colectomy [13].

In this study, we investigated three cases of internal hernia passing through the defect around the preserved SRA after laparoscopic left-sided colectomy. We assessed the patient characteristics and space between the SRA and retroperitoneum after laparoscopic left-sided colectomy.

## 2 Methods

### 2.1 Patient selection and treatment

In this retrospective study, we enrolled 308 consecutive patients who underwent either laparoscopic left hemicolectomy or sigmoidectomy between July 2013 and February 2018 at the University of Tokyo Hospital. Data pertaining to the following variables were collected from the medical records: age, sex, body height and weight, body mass index (BMI), indication for surgery (malignant tumor or non-malignant disease such as diverticulitis and fistula), lesion site, SRA preservation, splenic flexure mobilization, and colostomy construction. The study was approved by the Ethics Committee of the University of Tokyo [No. 3252-(7)]. This article has been reported in line with the STROCSS criteria [14].

### 2.2 Surgical technique

Laparoscopic left-sided colectomy was performed using a medial-to-lateral approach in all patients with malignant disease. For patients with non-malignant disease, either a medial-to-lateral approach or lateral-to-medial approach was adopted based on the individual case characteristics and the preference of the surgeon [15].

For cancer located at the descending colon or sigmoid colon close to the sigmoid-descending colon junction, lymph nodes around the inferior mesenteric artery (IMA) were dissected, and the feeding artery was ligated at its origin; vascular flow of the IMA was preserved to maintain blood supply to the distal sigmoid colon [16].

For cancer located at the distal sigmoid colon, the origin of the IMA was ligated to achieve central vascular ligation [17].

For most non-malignant diseases, peripheral arteries in proximity to the intestinal tract were ligated. In patients with mesenteric abscess, the feeding artery was ligated at its origin. In patients with benign disease located close to the rectosigmoid colon, the SRA was ligated using a medial-to-lateral approach.

Colostomy construction without anastomosis was performed at the operator's discretion based on the evaluation of risk factors for anastomotic leakage, such as obstructive colitis and developing peritonitis.

No mesenteric defects were closed without placing absorbable adhesion barriers in all patients.

### 2.3 Measurement of the defect behind the SRA

**Fig. 1:**
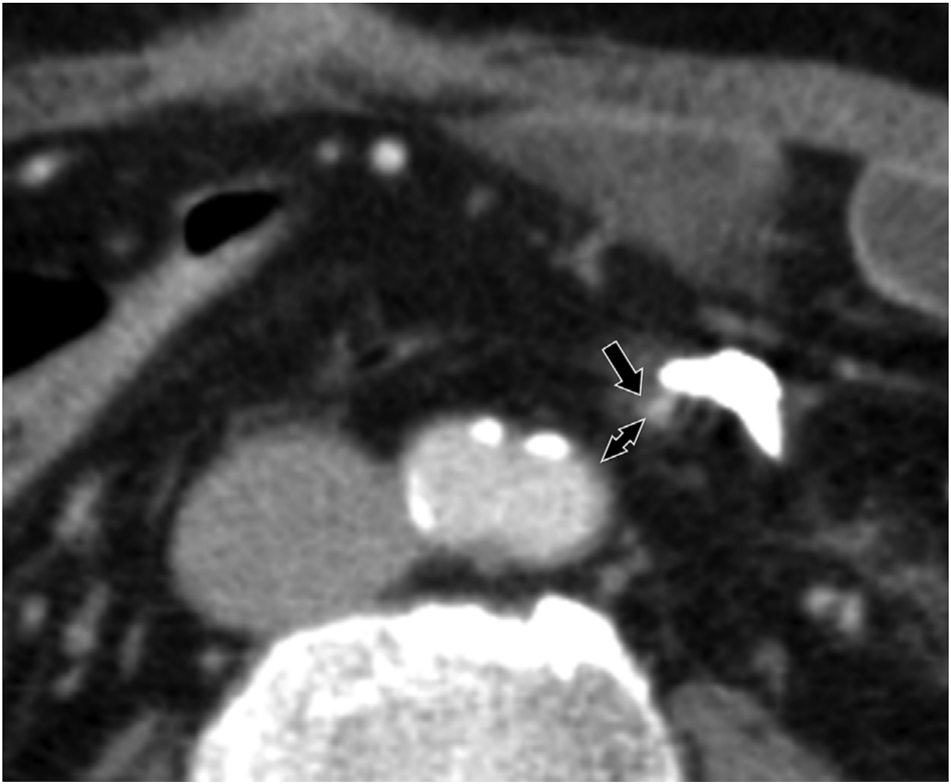
Distance between the superior rectal artery (SRA; arrow) and abdominal aorta at the aortic bifurcation level (double-headed arrow) was measured on computed tomography (CT) images obtained 3–6 months after SRA-preserving surgery.

All patients underwent enhanced computed tomography (CT) with 1–5 mm slice intervals 3–6 months after surgery for the first time. In patients who underwent SRA-preserving colectomy, the distance between the SRA and abdominal aorta at the level of the aortic bifurcation was measured using an axial image of the postoperative CT (Fig. 1).

### 2.4 Statistical analysis

Unpaired *t*-test was used to compare the distance between the SRA and abdominal aorta between ostomy patients and non-ostomy patients. All analyses were performed with the JMP Pro 14.0 software (SAS Institute Inc, Cary, NC); p-values < 0.05 were considered to indicate statistical significance.

## 3 Results

### 3.1 Frequency of internal hernia

The median follow-up period of the 308 patients enrolled in this study was 33.2 months. Three patients (0.97%) developed symptomatic internal hernia. Table 1 summarizes the details of the 3 patients. One patient responded to conservative treatment (Case 1), while the other 2 patients developed small bowel strangulation, which necessitated re-operation (Cases 2 and 3). Case 3 developed irreversible bowel ischemia and underwent bowel resection (Case 3); the clinical course of this patient is presented later.

**Table 1:** Summary of the three patients who developed symptomatic internal hernia.

Patients No, age (years)/sex	Location of the resected lesion	Interval from the initial colectomy	Management
1. 54/M	Sigmoid colon	16 days	Conservative
2. 84/F	Sigmoid colon	8 months	Re-operation without surgical bowel resection
3. 73/M	Sigmoid colon	2 months	Re-operation with surgical bowel resection

F: female, M: male, SRA: superior rectal artery.

### 3.2 Correlation between internal hernia and clinical characteristics

The clinical characteristics and incidence of internal hernia are shown in Table 2. 3.0% of patients who preserved SRA and 21.4% of patients who construct colostomy developed internal hernia.

**Table 2:** Clinical characteristics and development of internal hernia.

	Internal hernia	
	Negative (n = 305)	Positive (n = 3)
**Gender**		
Male	174 (98.9%)	2 (1.1%)
Female	131 (99.2%)	1 (0.8%)
**Age, years**		
≤65	142 (99.3%)	1 (0.7%)
>65	163 (98.8%)	2 (1.2%)
**BMI, kg/m**^**2**^		
≤25	237 (99.2%)	2 (0.8%)
>25	68 (98.6%)	1 (1.4%)
**Indication for surgery**		
Malignant tumor	294 (99.7%)	1 (0.3%)
Non-malignant disease	11 (84.6%)	2 (15.4%)
**Site of lesion**		
Descending colon	56 (100%)	0 (0%)
Sigmoid colon	249 (98.8%)	3 (1.2%)
**SRA preservation**		
Preserved	98 (97.0%)	3 (3.0%)
Transected	207 (100%)	0 (0%)
**Splenic flexure mobilization**		
Yes	71 (100%)	0 (0%)
No	234 (98.7%)	3 (1.3%)
**Construction of stoma**		
Yes	11 (78.6%)	3 (21.4%)
No	294 (100%)	0 (0%)

BMI: body mass index, SRA: superior rectal artery.

### 3.3 Postoperative evaluation of the defect behind the SRA

**Fig. 2:**
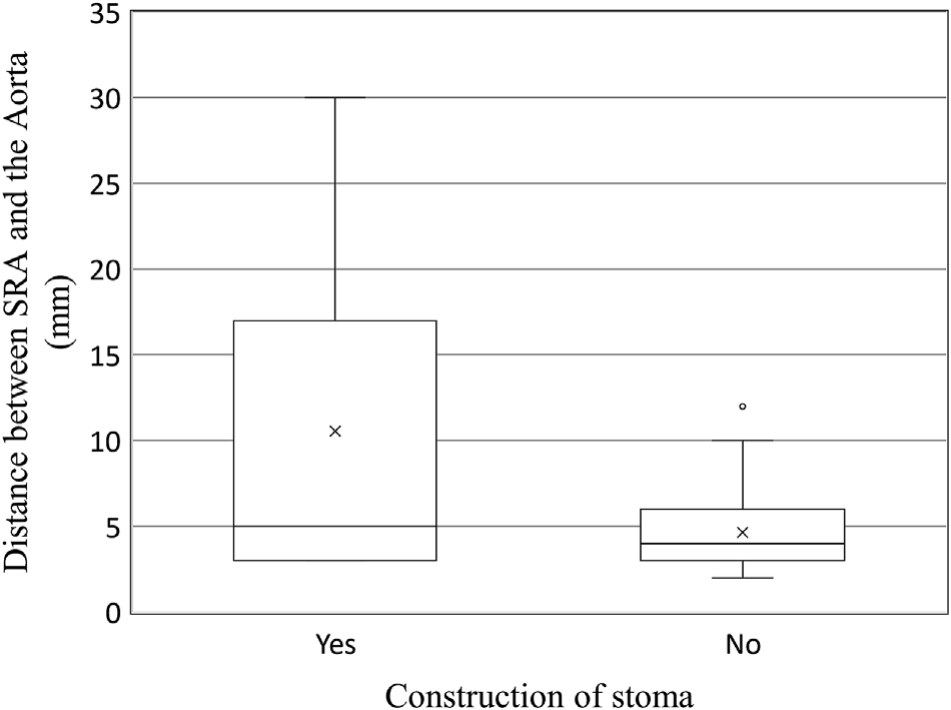
Distance between the superior rectal artery (SRA) and abdominal aorta at the aortic bifurcation level. Ostomy patients had a larger space behind the SRA than non-ostomy patients (p < 0.001).

The distance between the SRA and abdominal aorta was analyzed with regard to stoma construction in patients who underwent SRA-preserving colectomy (Fig. 2). The evaluation was performed after a median period of 4.6 months post-surgery. The distance was significantly longer in the ostomy patients than in the non-ostomy patients (10.6 mm vs. 4.7 mm, p < 0.001). Specifically, the mean distance was 16.5 mm in the 2 ostomy patients who required re-operation.

### 3.4 Case presentation

**Fig. 3:**
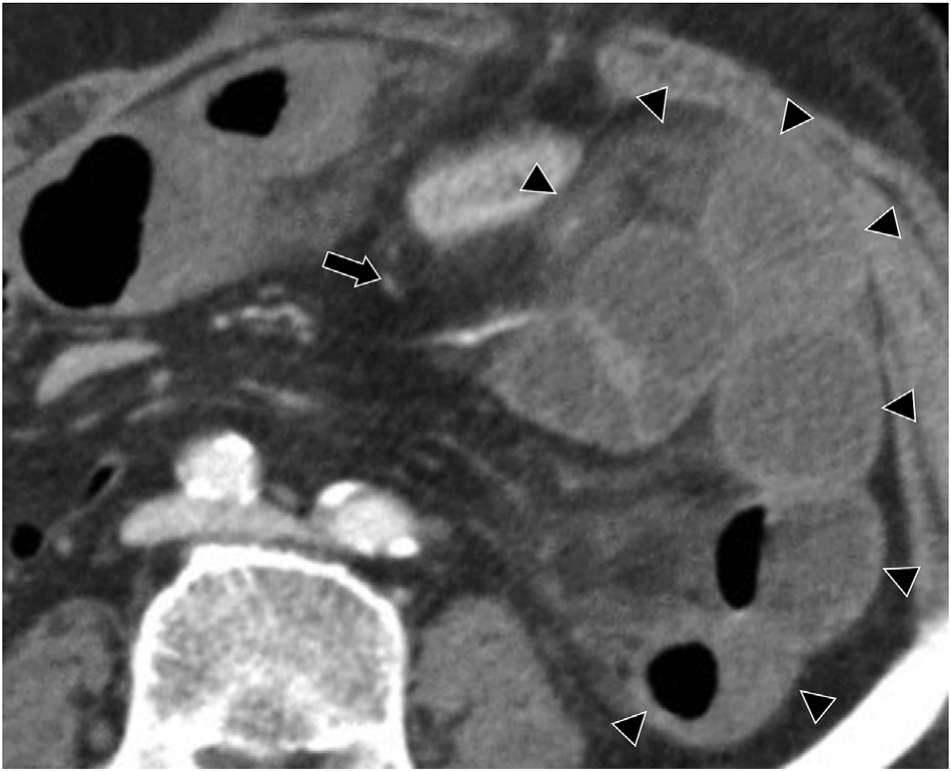
Axial computed tomography (CT) of Case 3 showing dilated and edematous small bowel (arrowheads). It also shows passage of the small bowel through the defect behind the superior rectal artery (SRA; arrow).

**Fig. 4:**
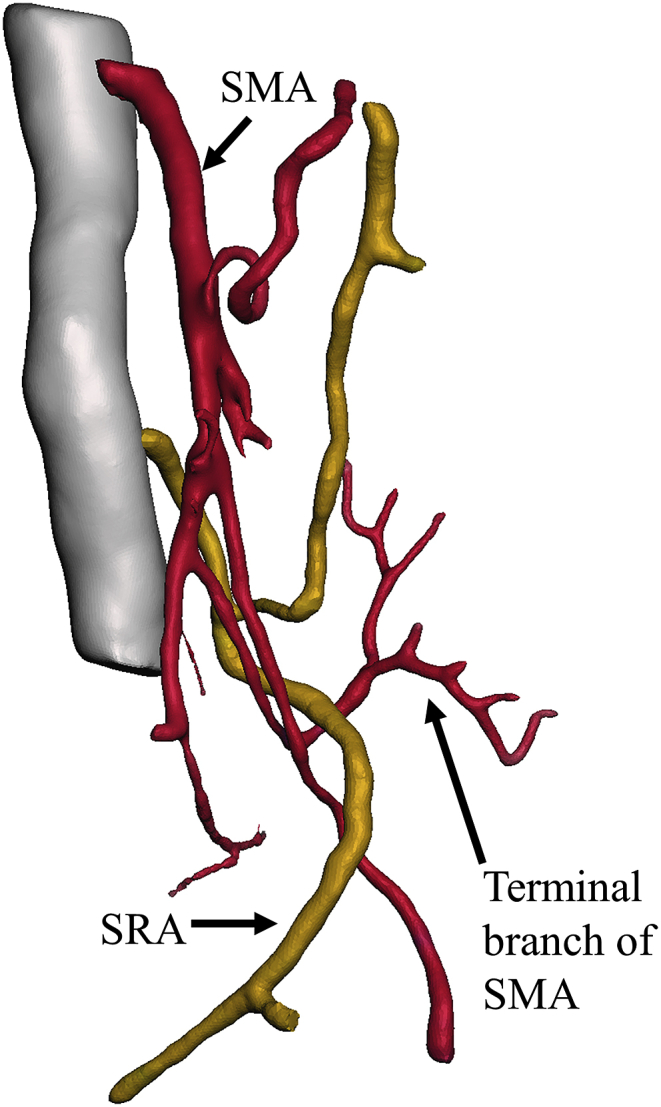
3D computed tomography (CT) angiogram of Case 3 showing a branch of the superior mesenteric artery (SMA) passing through the space behind the superior rectal artery (SRA). Red: SMA; yellow: SRA. . (For interpretation of the references to colour in this figure legend, the reader is referred to the Web version of this article.)

A 73-year-old man with a history of long-term steroid therapy (prednisolone 70 mg/day) for polymyositis developed colon cancer located at the sigmoid-descending colon junction. He underwent laparoscopic sigmoidectomy with SRA preservation. A double-barreled colostomy was constructed without anastomosis. Two months after the operation, the patient developed severe acute abdominal pain. CT revealed a closed loop of small bowel with dilation and edematous change suggesting strangulation, because the segment passed through the defect behind the SRA (Fig. 3). This finding was also observed on CT angiography (Fig. 4).

**Fig. 5:**
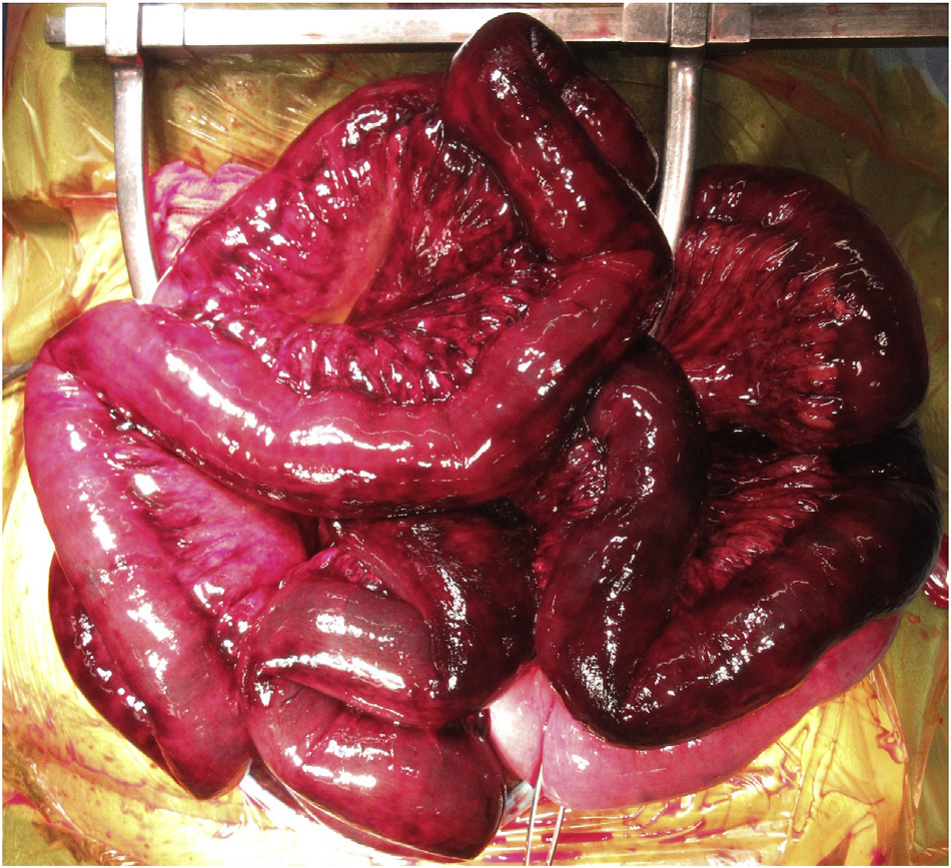
Intraoperative photograph of Case 3 showing a congested and ischemic segment of the strangulated ileum requiring resection.

Laparotomy revealed strangulation of a 160 cm segment of the small bowel passing through the defect behind the SRA. Because the congested segment showed no recovery after relief of strangulation (Fig. 5), partial resection of the small bowel was performed with closure of the mesocolonic defect. There were no complications or recurrence of internal hernia after the re-operation.

## 4 Discussion

The incidence of internal hernia after laparoscopic left-sided colectomy in the present study was 0.97% (3/308). In a meta-analysis, the incidence of internal hernia after laparoscopic colectomy was 0.65% (25/3813) [8], which is similar to our data.

In this study population, 9 patients underwent colectomy without anastomosis, while SRA was preserved. Among these, 3 patients (33.3%) developed internal hernia. We presume that the SRA was levitated ventrally by the colostomy fixation to the abdominal wall. In the present study, the mean distance between the SRA and abdominal aorta among non-ostomy patients who underwent SRA-preserving surgery was only 4.7 mm. In contrast, the mean distance between the SRA and abdominal aorta in ostomy patients was 10.6 mm. In a study by Martin et al. small size of mesenteric defects (2–5 cm) was found to be a risk factor for volvulus and strangulation of the herniated loops of the bowel [18]. In fact, the mean defect size in our 2 patients who required surgical intervention for internal hernia was 16.5 mm.

In a meta-analysis (combined n = 881), medial-to lateral approach for laparoscopic left-sided colectomy was associated with shorter operative time and a lower conversion rate than the lateral-to-medial approach [19]. Therefore, for this procedure, the medial-to-lateral approach was recommended by the European Association of Endoscopic Surgeons [20], although this approach inevitably creates a space behind the SRA.

This study had some limitations. First, this was a retrospective study that included a very small number of events; thus, we could not draw any definitive conclusions. Secondly, we evaluated only patients who underwent postoperative CT; therefore, the incidence of internal hernia may have been underestimated. Moreover, internal hernia without small bowel obstruction or symptoms was not evaluated.

SRA preservation and colostomy construction without anastomosis could be considered as risk factors for internal hernia after laparoscopic left-sided colectomy.

During laparoscopic surgery with SRA preservation via the medial-to-lateral approach, it is important to ensure that the size of the defect behind the SRA is narrow enough to avoid internal hernia. Moreover, given the increased incidence of internal hernia associated with the medial-to-lateral approach, a lateral-to-medial approach should be considered, especially for laparoscopic surgery for non-malignant disease or when colostomy is planned.

## Ethical approval

All procedures performed in studies involvingv human participants were in accordance with the Ethics Committees of the University of Tokyo [No. 3252-(7)].

## Sources of funding

This research is supported by Grants-in-Aid for Scientific Research (C: grant number; 17K10620, C: grant number; 17K 10621, C: grant number; 17K10623, C: grant number; 18K07194, C: grant number; 19K09114, C: grant number; 19K09115) from Japan Society for the Promotion of Science. This research is supported by the Project for Cancer Research and Therapeutic Evolution (P-CREATE), grant number: 18cm0106502h0003 from the Japan Agency for Medical Research and Development.

## Author contribution

TT wrote the manuscript. TT, KM, HN, DH, KK, KH, TT, TN, KS, YS, MK, SE, HS, and SI acquired the clinical and pathological data. DH contributed to make 3D computed tomography (CT) angiogram. KM and HN contributed to editing the manuscript. All authors read and approved the final manuscript.

## Consent

Informed consent was documented in the paper in all patients.

## Registration of research studies

1. Name of the registry: the Research Registry
2. Unique Identifying number or registration ID: 4979
3. Hyperlink to the registration (must be publicly accessible): https://www.researchregistry.com/browse-the-registry#home/

## Guarantor

Tetsuro Taira.

## Provenance and peer review

Not commissioned externally peer reviewed.

## Declaration of competing interest

There are no conflicts of interest.

## Appendix A Supplementary data

the_strocss_statement_checklist the_strocss_statement_checklist
